# MACI “Sandwich” Technique for a Large Osteochondritis Dissecans Lesion: A Case Report

**DOI:** 10.1155/2023/7612206

**Published:** 2023-02-15

**Authors:** Bhumit Desai, Graylin Jacobs, Deryk Jones

**Affiliations:** ^1^Department of Orthopaedic Surgery, Ochsner Medical Center, 1514 Jefferson Hwy, Jefferson, LA 70121, USA; ^2^Ochsner Sports Medicine Institute, 1201 S. Clearview Pkwy, New Orleans, LA 70121, USA

## Abstract

There is widespread use of matrix-induced autologous chondrocyte implantation. Initial use of autologous bone grafting in combination with the matrix-induced autologous chondrocyte implantation procedure has shown efficacy in small- to medium-sized osteochondral lesions. This case report demonstrates use of the “Sandwich” technique in a large, deep osteochondritis dissecans lesion of the medial femoral condyle. Technical considerations that are key to containment of the lesion and outcomes are reported.

## 1. Introduction

**Figure 1:**
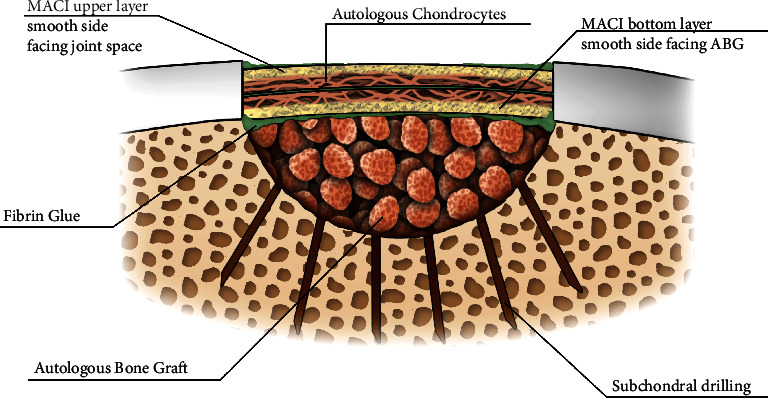
Bone graft is placed up to the subchondral plate level. Base drilled with 1.5 mm drill bit.

Lars Peterson described placing autologous periosteum above and below implanted chondrocytes with concomitant autologous bone grafting (ABG) up to the subchondral bone plate level to treat osteochondritis dissecans (OCD) lesions [1]. This technique was referred to as the “Sandwich” technique (Figure 1).

Demange et al. compared the “Sandwich” technique to ABG alone with a graft survival rate of 87% in the autologous chondrocyte implantation (ACI) “Sandwich” group and 54% in the ABG group [2]. Garrett et al. reported on OCD lesions treated with osteochondral allografts (OCA) but fragmentation and poor bone incorporation occurred in a lesion sized at 3.0 × 4.5 cm [3]. Osteochondral allografts contain donor human leukocyte antigens causing rejection of grafts limiting the amount of donated bone that can be implanted [4].

A porcine bilayer type I–III collagen membrane was developed as a chondrocyte carrier leading to the third-generation of ACI—autologous cultured chondrocytes on porcine collagen membrane (MACI) [5, 6]. This case demonstrates treatment of a large deep condylar OCD lesion with high tibial osteotomy (HTO), ABG, and the bilayer MACI “Sandwich” technique. Technical factors important to appropriate healing of the bone defect in combination with methods to control bleeding, contain the lesion, and maintain support for gradual load-bearing post-operatively are discussed.

## 2. Case Presentation

### 2.1. Patient Information

**Figure 2:**
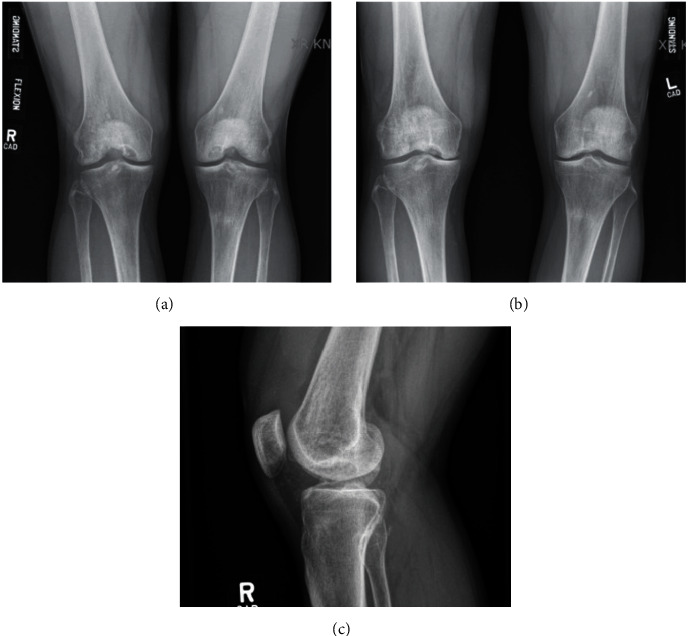
Preoperative radiographs. (a) Posterior-anterior weight-bearing view. (b) Anterior-posterior weight-bearing view. (c) Lateral view.

An active 34-year-old male presented with bilateral symptomatic medial OCD lesions. Body-mass index was 30 kg/m^2^ with genu varum, no muscular atrophy, and mild effusions bilaterally. Range of motion (ROM) was 0° of hyperextension, 0° extension, and 135° flexion bilaterally. Menisci and ligamentous examinations were normal, with medial femoral condyle pain on palpation in the right greater than the left knee. Preoperative radiographs demonstrated underlying genu varum with large medial femoral OCD lesions and a right knee Kellgren–Lawrence (KL) grade 2 and left knee KL grade 1 (Figures 2(a), 2(b), and 2(c)).

**Figure 3:**
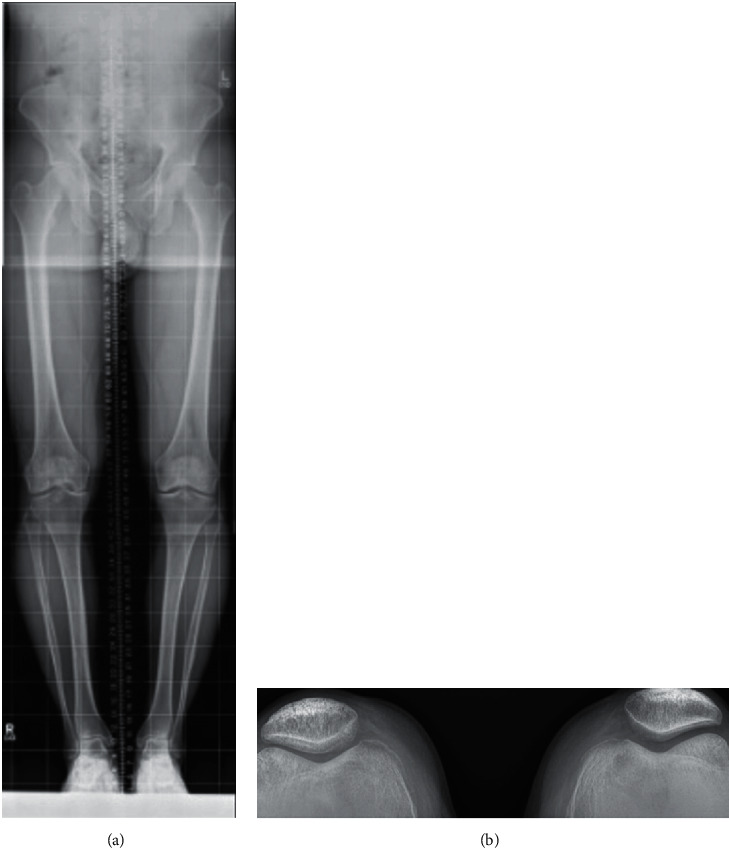
(a) Hip-to-ankle views. (b) Merchant's patellofemoral view.

Hip-to-ankle radiographs demonstrated femoral-tibial angles of 8° on the right and 3° on the left (Figure 3(a)). There was no patellofemoral involvement on Merchant's views (Figure 3(b)).

**Figure 4:**
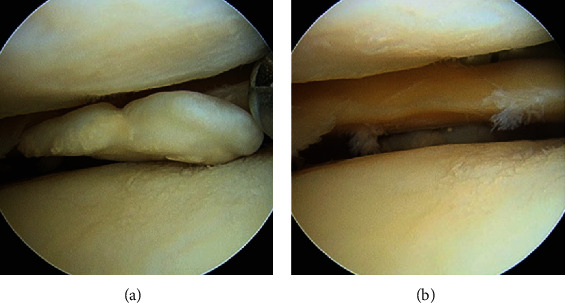
Arthroscopy. (a) Loose body removal. (b) Lateral compartment view.

No preoperative magnetic resonance imaging (MRI) was available due to work-related issues and timing. The patient decided to proceed with diagnostic arthroscopy and if indicated, planned biopsy from the intercondylar notch. At arthroscopy, a loose body was removed from the lateral compartment (Figures 4(a) and 4(b)).

**Figure 5:**
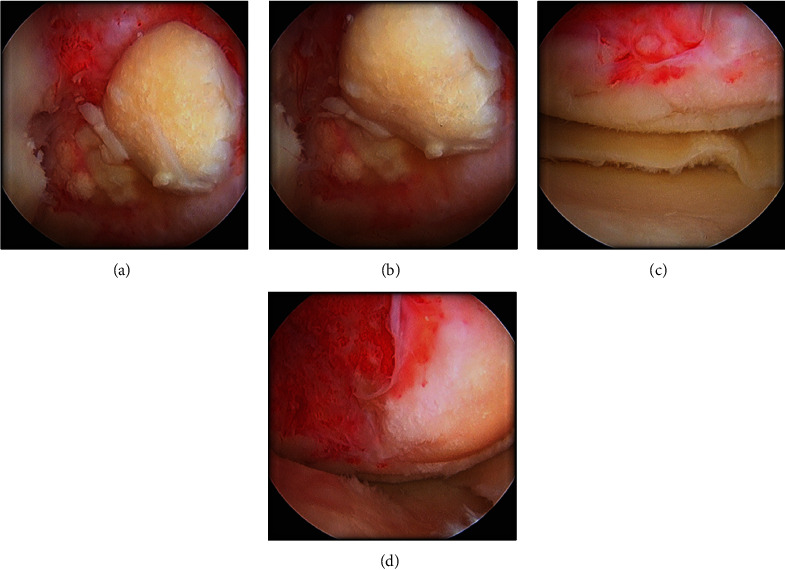
Arthroscopy. (a) Fragmentation medial OCD. (b) Irreparable fragment. (c) Medial posterior OCD. (d) Bone defect/medial wear.

A large, irregular OCD lesion with a femoral chondral fragment with necrotic bone and medial tibial wear was seen in the medial compartment (Figures 5(a), 5(b), 5(c), and 5(d)).

**Figure 6:**
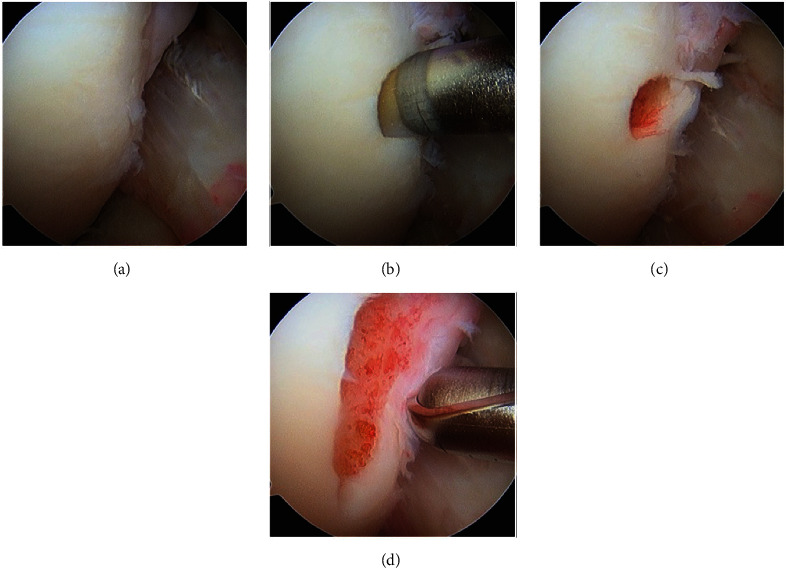
Cartilage biopsy. (a) Lateral intercondylar notch. (b) Needle biopsy. (c) Initial biopsy site. (d) Completed biopsy.

An unstable irreparable osteochondral fragment was removed, and pathology assessment revealed a cartilaginous fragment with osteonecrotic bone base (2.0 × 1.5 × 0.5 cm). Intra-operative measurements demonstrated a lesion size of 3.0 × 4.5 cm with a bony depth of 2 cm. A cartilage biopsy from the right knee intercondylar notch was obtained (Figures 6(a), 6(b), 6(c), and 6(d)), and due to the large defect size, two MACI membranes were automatically ordered.

Based on the preoperative work-up, diagnostic arthroscopy, bony depth, and lesion size, the decision was made to proceed with right knee MACI “Sandwich” technique with concomitant HTO followed by left knee surgery, allowing adequate time for recovery.

### 2.2. Surgical Procedure

**Figure 7:**
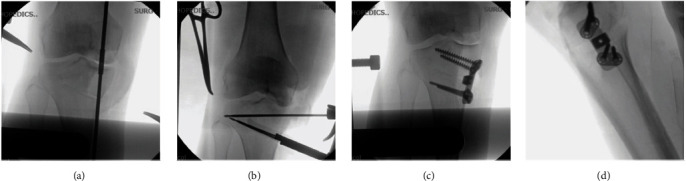
High tibial osteotomy X-rays. (a) Intra-operative mechanical axis. (b) Alignment HTO guide. (c) HTO fixation anterior view. (d) HTO fixation lateral view.

**Figure 8:**
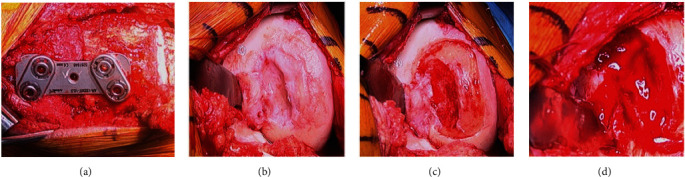
HTO, lesion characteristics. (a) High tibial osteotomy. (b) OCD poorly contained lesion. (c) Prepared lesion site. (d) Bleeding created base lesion.

A 6 cm anteromedial subvastus approach and HTO with 10° correction were performed (Figures 7(a), 7(b), 7(c), 7(d), and 8(a)).

A large, poorly contained lesion measuring 3 × 4.5 cm was demonstrated (Figure 8(b)). The periphery of the lesion was demarcated with a # 15 blade, and ringed curettes were used to remove damaged cartilage to normal vertical borders. A shelf was created between normal subchondral bone plate level and bone defect. Sclerotic bone and intralesional osteophytes were removed with a high-speed burr creating a healthy bleeding base (Figure 8(c)). The site was drilled with a 1.5 mm drill bit at 3 mm increments to increase blood flow into the lesion (Figure 8(d)).

**Figure 9:**
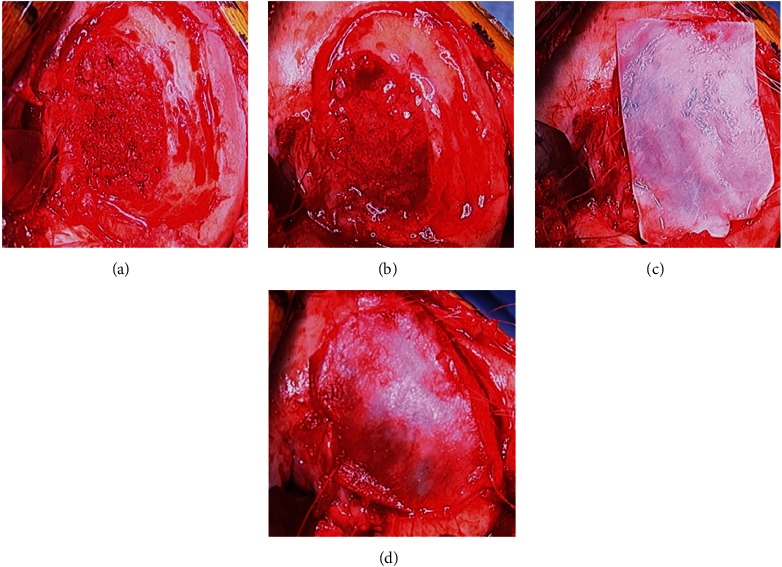
Bone grafting and first MACI stabilization. (a) Bone graft defect. (b) Fibrin glue base lesion. (c) MACI graft # 1 cell layer up. (d) MACI graft # 1 suture stabilization.

Iliac crest ABG was obtained, and bone impacted into the defect up to, but not above, the subchondral bone plate level demarcated by the previously created shelf (Figure 9(a)). Three absorbable micro-anchors were placed along the poorly contained region of the intercondylar notch. A Keith needle was used to create osseous tunnels incrementally along the medial border of the lesion. The lesion required the entire MACI graft. Fibrin sealant was placed over the base of the lesion (Figure 9(b)). The first graft was placed smooth-side facing towards the bone graft and sewn into position using the micro-anchors along the intercondylar notch region (Figure 9(c)); additional 5-0 vicryl sutures were placed using the osseous tunnels previously created with the Keith needle, and 6-0 vicryl sutures were placed proximally and distally at the normal articular cartilage margins (Figure 9(d)).

**Figure 10:**
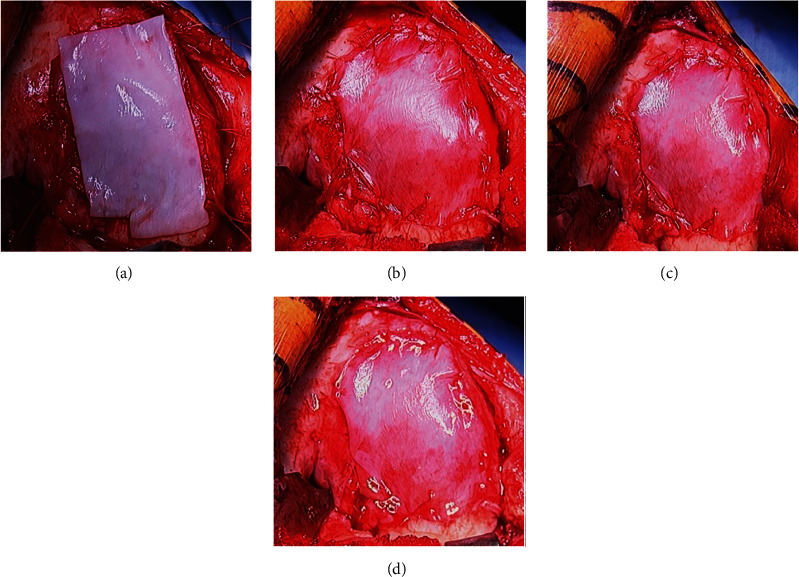
Second MACI stabilization. (a) MACI graft # 2 cell layer down. (b) MACI graft # 2 suture stabilization. (c) MACI graft # 2 sewn. (d) MACI grafts sewn with fibrin glue.

The second graft was placed with the cell layer facing into the defect and sewn into position using the same sutures (Figures 10(a), 10(b), and 10(c)). Fibrin sealant was placed around the periphery of the lesion (Figure 10(d)). Standard closure was performed.

### 2.3. Rehabilitation Program

Continuous passive motion (CPM) machine with ROM to 30° flexion increasing by 15° per week was started on post-operative day (POD) # 1 with full active-assisted ROM initiated and CPM machine discontinued at 4 weeks; full ROM and immobilization were discontinued at 6 weeks. Toe-touch weight-bearing was allowed locked in extension on POD # 1, 25% partial weight-bearing (PWB) at 2 weeks, 25–50% PWB at 4 weeks, and full weight-bearing as tolerated at 6 weeks. Exercycle, elliptical, and core muscle strength programs were initiated at 6–8 weeks.

### 2.4. Follow-Up and Outcomes

**Figure 11:**
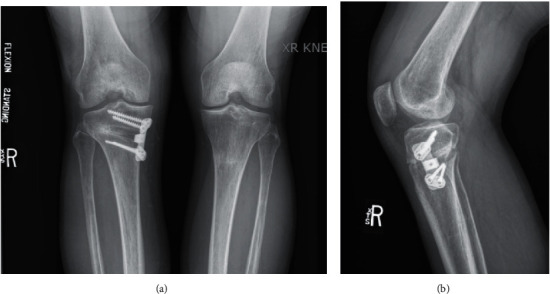
(a) 3-month post-operative posterior-anterior (PA) weight-bearing (WB) view. (b) 3-month post-operative lateral view.

**Figure 12:**
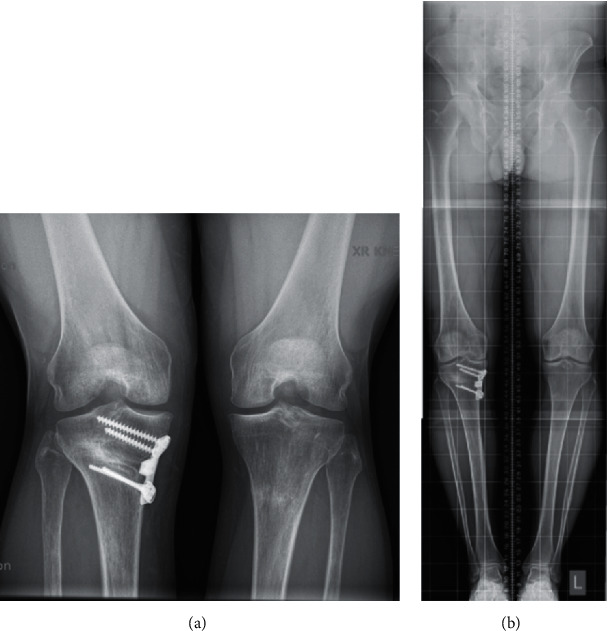
(a) 6-month post-operative PA WB view. (b) 6-month hip-to-ankle WB view.

Post-operative radiographs at 3 (Figures 11(a) and 11(b)) and 6 months (Figures 12(a) and 12(b)) revealed improved medial joint space, appropriate contour, fill, and bone incorporation.

**Figure 13:**
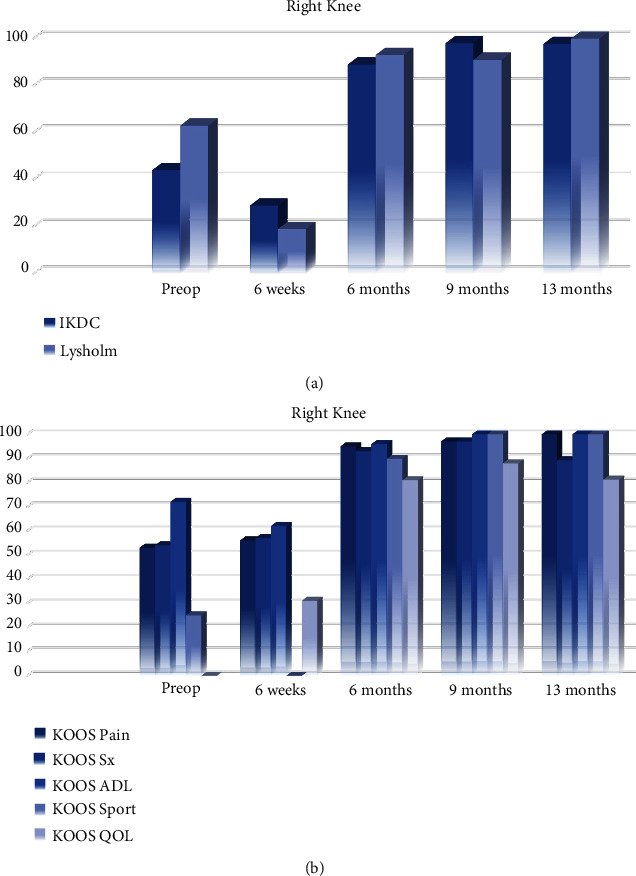
(a) Right knee IKDC and Lysholm. (b) Right knee KOOS.

All patient-reported outcome measures (PROMs) significantly improved from baseline at 13 months. Lysholm and International Knee Documentation Committee (IKDC) scores improved by 37 and 54 points, respectively (Figure 13(a)). Knee Injury and Osteoarthritis Outcome Score (KOOS) pain and symptom scores improved 47 and 35 points, respectively, while KOOS sports improved by 75 points and quality of life increased by 81 points at the 13-month follow-up (Figure 13(b)). The PROMs delta augmentation prevails over the minimum clinically important difference (MCID).

**Figure 14:**
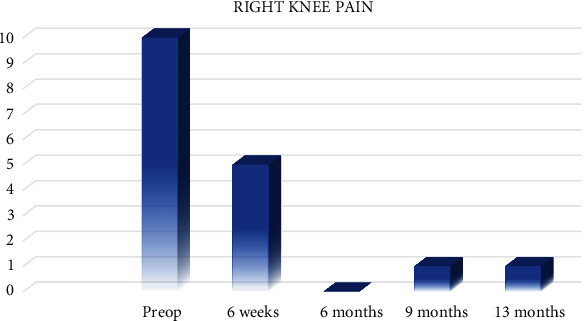
Right knee pain VAS.

Left knee pain improved as right knee pain improved (Figure 14).

**Figure 15:**
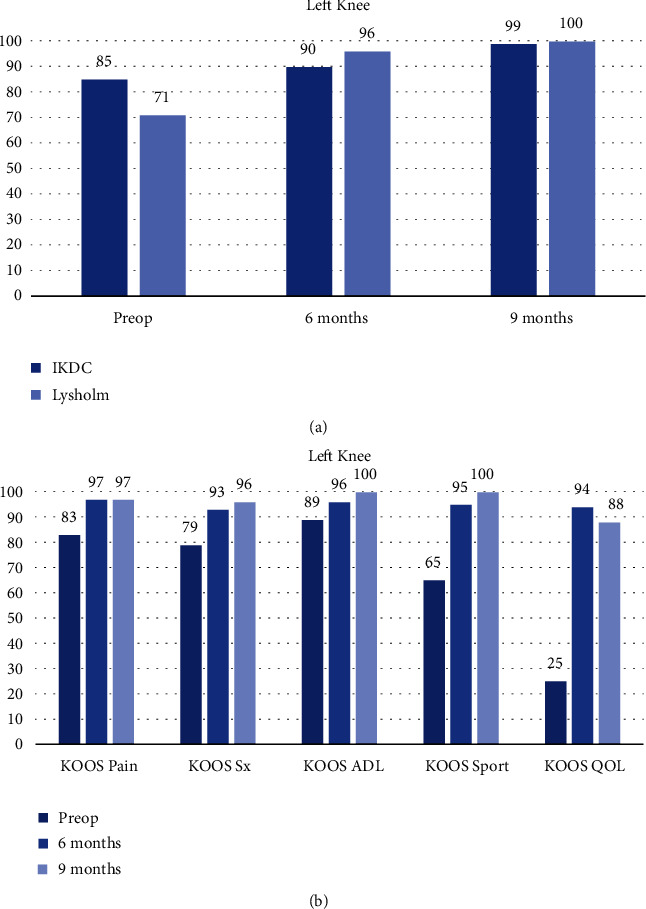
(a) Left knee IKDC and Lysholm. (b) Left knee KOOS.

**Figure 16:**
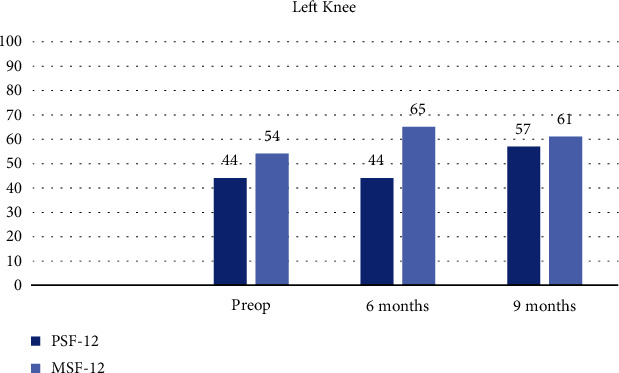
Patient short form and multisource feedback.

The left knee PROMs stabilized (Figures 15(a) and 15(b)) and PSF-12 measurements improved by 21 points at 9 months (Figure 16).

## 3. Discussion

The MACI “Sandwich” procedure with concomitant bone grafting and realignment surgery is effective, as previously reported with the ACI “Sandwich” technique. ABG is preferred and can be obtained from proximal tibia or iliac crest [7]. The autologous patient's bone and chondrocytes participate in developing the blood flow pattern and graft incorporation [1, 8]. Ogura reported outcomes in patients treated with the “Sandwich” technique (periosteum and ACI), but no lesions were treated with third-generation MACI membranes [8].

Feedback from surgeons using the MACI graft in combination with the “Sandwich” technique shows no detrimental effects [9]. ACI segmental-sandwich technique provides excellent survival rates at midterm follow-up by restoring congruence and providing native joint preservation. Although one or two MACI membranes can be used and have been discussed in the literature, both methods for the treatment of OCD lesions have been described, with a recommendation for using two membranes, especially for large osteochondral lesions, to secure the underlying ABG [7, 9].

One graft was used with the cell layer facing the bone defect and fibrin glue at the base in small- to medium-sized well contained lesions as described by Jones and Cash for a small osteochondral defect [7]. In large, poorly contained lesions, suture fixation and two-layer graft technique mimicking the original “Sandwich” technique seems appropriate. This stabilizes the bleeding of the lesion while containing the area and sealing it from the intra-articular space to retain chondrocytes within the defect area, consistent with previous instruction [10]. The adjacent tibial surface and joint motion contour the femoral lesion during the healing process, providing a remarkable appearance radiographically at 6–12 months post-operatively.

Containment and size are not contraindications in surgical procedures and may be managed using multiple MACI membranes with appropriate fixation. Suture anchor and suture-to-bone fixation stabilized the graft, sealed the defect along uncontained regions, and gave the patient and surgeon confidence during post-operative rehabilitation. This technique can be used as a salvage procedure in the case of a failed osteochondral autologous transplantation (OAT), OCA, or OCD fixation procedure as well. The MACI graft expedites procedure time while providing a homogenous population of functional chondrocytes. Good-to-excellent results were demonstrated using the MACI procedure with concomitant ABG of OCD lesions, but the clinical benefit decreased for defects exceeding 6 cm^2^ [11]. This proposed approach avoids rejection at the bone level, limiting late subchondral bone marrow lesion-related symptoms [12]. MRI is helpful in assessing the depth of bone involvement greater than 6.5 mm [13].

## 4. Conclusion

Based on the available information, no previously reported case combines the complexity of an HTO with ABG to treat an OCD lesion and large condylar defect using a bilayer MACI “Sandwich” technique. Post-operative Lysholm and IKDC scores improved significantly from preoprative scores at 9 and 13 months. All post-operative PROMs improved with KOOS ADL and Sports scores of 100 at 9 and 13 months. MACI combined with corrective osteotomy is a feasible treatment option for active patients that present with malalignment and large poorly contained condylar lesions with bony involvement.

## Data Availability

The datasets used and analyzed during the current study are available from the corresponding author upon reasonable request.

## Abbreviations

ABG: Autologous bone grafting
ACI: Autologous chondrocyte implantation
CPM: Continuous passive motion
HTO: High tibial osteotomy
IKDC: International Knee Documentation Committee
KOOS: Knee Injury and Osteoarthritis Outcome Score
MACI: Autologous cultured chondrocytes on porcine collagen membrane; matrix-induced chondrocyte implantation
MCID: Minimum clinically important difference
MRI: Magnetic resonance imaging
MSF: Multisource feedback
OAT: Osteochondral autologous transplantation
OCD: Osteochondritis dissecans
PA: Posterior-anterior
POD: Post-operative day
PROMs: Patient-reported outcome measures
PSF: Patient short form
PWB: Partial weight-bearing
ROM: Range of motion
VAS: Visual analog scale
WB: Weight-bearing.
